# Study of gray matter atrophy pattern with subcortical ischemic vascular disease-vascular cognitive impairment no dementia based on structural magnetic resonance imaging

**DOI:** 10.3389/fnagi.2023.1051177

**Published:** 2023-02-06

**Authors:** Lin Tan, Jian Xing, Zhenqi Wang, Xiao Du, Ruidi Luo, Jianhang Wang, Jinyi Zhao, Weina Zhao, Changhao Yin

**Affiliations:** ^1^Department of Neurology, Hongqi Hospital Affiliated to Mudanjiang Medical University, Mudanjiang, China; ^2^Department of Rehabilitation, The Sixth Affiliated Hospital of Harbin Medical University, Harbin, China; ^3^Department of Imaging, Hongqi Hospital Affiliated to Mudanjiang Medical University, Mudanjiang, China; ^4^Heilongjiang Key Laboratory of Ischemic Stroke Prevention and Treatment, Mudanjiang, China

**Keywords:** vascular cognitive impairment no dementia, subcortical ischemic cerebrovascular disease, structural magnetic resonance imaging, gray matter atrophy, dementia, neurodegeneration

## Abstract

**Objective:**

This study explored the structural imaging changes in patients with subcortical ischemic vascular disease (SIVD)-vascular cognitive impairment no dementia (VCIND) and the correlation between the changes in gray matter volume and the field of cognitive impairment to provide new targets for early diagnosis and treatment.

**Methods:**

Our study included 15 patients with SIVD-normal cognitive impairment (SIVD-NCI), 63 with SIVD-VCIND, 26 with SIVD-vascular dementia (SIVD-VD), and 14 normal controls (NC). T1-weighted images of all participants were collected, and DPABI and SPM12 software were used to process the gray matter of the four groups based on voxels. Fisher’s exact test, one-way ANOVA and Kruskal-Wallis H test were used to evaluate all clinical and demographic data and compare the characteristics of diencephalic gray matter atrophy in each group. Finally, the region of interest (ROI) of the SIVD-VCIND was extracted, and Pearson correlation analysis was performed between the ROI and the results of the neuropsychological scale.

**Results:**

Compared to the NC, changes in gray matter atrophy were observed in the bilateral orbitofrontal gyrus, right middle temporal gyrus, superior temporal gyrus, and precuneus in the SIVD-VCIND. Gray matter atrophy was observed in the left cerebellar region 6, cerebellar crural region 1, bilateral thalamus, right precuneus, and calcarine in the SIVD-VD. Compared with the SIVD-VCIND, gray matter atrophy changes were observed in the bilateral thalamus in the SIVD-VD (*p* < 0.05, family-wise error corrected). In the SIVD-VCIND, the total gray matter volume, bilateral medial orbital superior frontal gyrus, right superior temporal gyrus, middle temporal gyrus, and precuneus were positively correlated with Boston Naming Test score, whereas the total gray matter volume, right superior temporal gyrus, and middle temporal gyrus were positively correlated with overall cognition.

**Conclusion:**

Structural magnetic resonance imaging can detect extensive and subtle structural changes in the gray matter of patients with SIVD-VCIND and SIVD-VD, providing valuable evidences to explain the pathogenesis of subcortical vascular cognitive impairment and contributing to the early diagnosis of SIVD-VCIND and early warning of SIVD-VD.

## 1. Introduction

Vascular cognitive impairment (VCI) is the second largest type of cognitive impairment worldwide after Alzheimer’s disease and the second most common cause of dementia (Smith, 2017). It mainly refers to a series of syndromes with mild to severe cognitive impairment symptoms caused by cerebrovascular disease and its associated risk factors (Iadecola, 2013). Due to the severity and irreversibility of vascular dementia (VD), VCI can be effectively prevented and treated for dementia, and effective early recognition and intervention are keys to reducing the incidence of VD (Le Couteur et al., 2017). VCI-no dementia (VCIND), the mildest stage of VCI, is also the most common form of VCI in the elderly, diagnosed in 2.4% of people aged ≥65 years. It is also associated with an increased mortality risk (Rockwood et al., 2000). Therefore, early and effective prevention and treatment of VCIND are necessary.

Subcortical ischemic vascular disease (SIVD) is an important cause of VCI, and cognitive impairment caused by SIVD is also the most homogeneous and common subtype of VCI. Approximately 20% of strokes worldwide are caused by SIVD (Wu et al., 2015). The diagnostic criteria for SIVD are based on magnetic resonance imaging (MRI) findings, including a large number of white matter lesions (WMLs) and multiple lacunar infarctions (LIs)(Chen Q. et al., 2020). Neuroimaging provides strong support for improving the diagnostic accuracy of specific cognitive disorders (Frantellizzi et al., 2020). MRI, as a multiparameter selection, high temporal spatial resolution, and noninvasive evaluation method, has gained significant attention for the early diagnosis of VCI. Currently, MRI is the best method for providing imaging evidence for patients with cognitive impairment (Biessels, 2016). Structural MRI (sMRI), as a type of MRI, can accurately measure the thickness, density, volume, and other morphological structural changes of the cerebral cortex to quantitatively identify lesions.

This study primarily aimed to investigate the differences in gray matter volume between healthy controls and patients with SIVD, including those with normal cognition, VCIND, and VD, using gray matter density analysis.

## 2. Materials and methods

### 2.1. Participants

The present study was approved by the ethics committee of Hongqi Hospital affiliated with Mudanjiang Medical College. All participants provided written informed consent after complete explanation of the procedure. All patients in the case group were from the memory outpatient department and ward of Neurology, Hongqi Hospital affiliated with Mudanjiang Medical College from September 2020 to October 2021 with memory loss as the chief complaint. All normal control (NC) participants were recruited from a normal community population.

The diagnosis of SIVD was established unanimously by two radiologists who independently evaluated the T1-weighted imaging, T2-weighted imaging, and fluid-attenuated inversion recovery (FLAIR) MR images visually without knowledge of the participants’ clinical profiles. According to the diagnostic criteria of Román et al. (2002), SIVD showed multiple subcortical LIs or extensive WMLs or both on head MRI.

All participants were divided into four groups after completing the Beijing version of the Montreal Cognitive Assessment (MoCA) and Clinical Dementia Rating scale (CDR) under the supervision of a physician, including 10 healthy controls (HCs, *n* = 14), patients with SIVD with normal cognitive impairment (SIVD-NCI, *n* = 15), patients with SIVD with VCI without dementia (SIVD-VCIND, *n* = 63), and patients with SIVD with VD (SIVD-VD, *n* = 26). For the MoCA (Nasreddine et al., 2005), the cutoff value for cognitive impairment was <26. In addition, one additional point was added to the raw MoCA score when the participant’s educational level was <12. All participants were right-handed Han people aged >45 years from northeast China. The criteria for HCs included the following: (1) no chief complaint of cognitive decline, (2) MoCA score ≥ 26 and CDR score = 0, (3) no positive symptoms or signs observed during physical examination, and (4) no evident abnormality found on head MRI. The inclusion criteria for the SIVD-NCI group were as follows: (1) no chief complaint of cognitive decline, (2) MoCA score ≥ 26 and CDR score = 0, and (3) SIVD observed on head MRI. The diagnosis of patients with SIVD-VCIND was established based on the International Society for Vascular Behavioral and Cognitive Disorders’ guidelines (Sachdev et al., 2014): (1) complaints of cognitive dysfunction or cognitive dysfunction provided by insiders and normal or slightly impaired ability to perform the activities of daily living, (2) MoCA score < 26 and CDR score = 0 or 0.5, and (3) cognitive impairment caused by SIVD. The diagnosis of patients with SIVD-VD was established based on the Diagnostic and Statistical Manual of Mental Disorders, Fifth Edition and International Workshop of the National Institute of Neurological Disorders and Stroke-Association Internationale pour la Recherche et l’Enseignement en Neurosciences criteria (Roman et al., 1993): (1) chief complaint of cognitive dysfunction or cognitive dysfunction provided by an insider and significantly impaired ability to perform the activities of daily living, (2) MoCA score < 22 and CDR score ≥ 1 and (3) cognitive impairment caused by SIVD.

Participants were excluded if they had one or more of the following: (1) heart or kidney failure, cancer, or other serious systemic diseases; (2) evidence of macrovascular lesions (lesion diameter > 15 mm or watershed infarction); (3) cognitive impairment caused by causes other than cerebrovascular diseases; (4) progressively aggravated memory impairment or other cognitive impairments, with no corresponding imaging changes; (5) severely impaired hearing, vision, and language and inability to communicate; (6) Hamilton Depression and Anxiety Scale showing depression and anxiety; (7) a history of mental illness or abnormal congenital development; and (8) inability or refusal to undergo brain MRI.

### 2.2. Clinical data collection

The following clinical information was collected from the participants: (1) demographic characteristics, including sex, age, and educational level; (2) history of hypertension, diabetes, or heart disease; (3) personal smoking and drinking history; (4) blood pressure (systolic and diastolic blood pressure levels of all participants were recorded on the day of MRI examination); and (5) venous blood collected from the forearm of all participants after fasting for 12 h (blood was sent to the Laboratory of Red Flag Hospital affiliated with Mudanjiang Medical College for routine blood and biochemical tests). The interval between blood collection and MRI for all participants was no more than 7 days.

### 2.3. Clinical cognitive assessment

All participants were administered a comprehensive neuropsychological assessment battery that included the following aspects: Auditory Verbal Learning Test-HuaShan version (AVLT-H) for episodic memory, Boston Naming Test (BNT) for language function, and Shape Trail Tests A and B (STT-A and STT-B) for executive functions, all of which were completed in a strict order in accordance with the standard protocols in a quiet room.

### 2.4. Acquisition of images

All participants were analyzed and scanned using a 3 Tesla Philips Achieva MRI scanner. A T2W-FLAIR sequence was used to detect WMLs. A standard T1-weighted three-dimensional magnetization prepared rapid gradient echo sequence was applied with the following parameters: field of view = 256 × 256 mm^2^, layer thickness = 1 mm, GAP = 0, layer number = 192, repetition time/echo time/inversion time = 7/3.2/1100 ms, turn angle = 7°, and matrix = 256 × 256. Rubber earplugs were used to reduce noise, and foam cushions were used to fix the participants’ heads to minimize potential motion artifacts.

### 2.5. Processing of images

We used Mricron^1^, SPM12^2^, and DPABI^3^ software package to analyze the NMR data and calculate the gray matter volume of the whole brain voxel. All the above are operated on MatLab (R2015b).

The following were the main steps: (1) MRIcron software was used to convert the MRI data DICOM files of all participants into NIfTI files; (2) the NIfTI files were imported into CAT12 in SPM12 for segmentation; (3) quality checks were performed, and the segmented gray matter image was smoothed; (4) the smoothed data were imported into DPABI for statistical analysis and image presentation (all gray matter structures were partitioned using Anatomical Automatic Labeling);. and (5) the significant brain region of the SIVD-VCIND group was set as the region of interest (ROI) using DPABI, and the gray matter volume of the ROI was obtained.

### 2.6. Statistical analyses

All the clinical and demographic data were evaluated among the groups using SPSS version 21. Count data are presented as case numbers (constituent ratios), and Fisher’s exact test was used to analyze the demographic data of the participants. Normally distributed data are represented as mean ± standard deviation, whereas non-normally distributed data are represented by M (Q1, Q3). The data with normal distribution were analyzed by one-way analysis of variance (ANOVA), and the least significant difference was used for *post hoc* tests. Non-normally distributed data were tested using the Kruskal–Wallis H test, an independent sample nonparametric test (*p* < 0.05 was considered statistically significant).

All MRI image data were analyzed using DPABI. ANOVA was used for comparison among the four groups; two-sample T-test was used for comparison between the two groups; age, sex, educational level, and total brain volume were used as covariables; and the permutation test was used for multiple comparison correction to analyze the atrophy changes of gray matter in the participants. The gray matter volume of SIVD-VCIND was extracted from DPABI, and Pearson’s correlation analysis was performed between SIVD-VCIND and neuropsychological scale results (*p* < 0.05 was considered statistically significant).

## 3. Results

### 3.1. Demographic and clinical data

A total of 118 participants were included in this study, and the participants’ demographic and clinical data, including age, sex, educational level, past history, personal history, and blood tests, were assessed. The results showed that there were statistically significant differences in age, educational level, hypertension, systolic blood pressure, and monocyte ratio among the four groups (*p* < 0.05). Systolic blood pressure in the SIVD-NCI group was higher than that in the NC group (p < 0.05). Compared with the NC group, the SIVD-VCIND group had a lower educational level and more patients with advanced age and a history of hypertension (*p* < 0.05). The SIVD-VD group had higher systolic blood pressure and monocyte ratio (*p* < 0.05), higher age than the SIVD-NCI group (*p* < 0.05), and higher systolic blood pressure than the SIVD-VCIND group (*p* < 0.05; see Table 1 for detailed data).

**Table 1:** Demographic and clinical characteristics of participants.

		NC (*n* = 14)	SIVD-NCI (*n* = 15)	SIVD-VCIND (*n* = 63)	SIVD-VD (*n* = 26)	*F/H/χ*^2^ value	*p* value
Gender	M	6 (42.86)	9 (60.00)	24 (38.10)	17 (65.38)	6.631	0.085
	F	8 (57.14)	6 (40.00)	39 (61.90)	9 (2.89)		
Age (years)		53.50 (47.50, 57.50)	56.00 (51.00, 64.00)	61.00 (56.00, 70.00)	67.00 (60.00, 74.00)	25.968	<0.001^b,c,e^
Education		11.00 (9.00, 12.00)	9.00 (8.00, 12.00)	8.00 (6.00, 9.00)	7.00 (4.00, 11.00)	12.829	0.005^b,c,^
Hypertension, *n* (%)		3 (21.43)	9 (60.00)	35 (55.56)	19 (73.08)	9.967	0.019^b,c^
Diabetes, *n* (%)		1 (7.14)	2 (13.13)	13 (20.63)	5 (19.23)	1.674	0.761
Cardiopathy, *n* (%)		2 (14.29)	0 (0)	7 (11.11)	5 (19.23)	3.481	0.289
Smoking, *n* (%)		4 (28.57)	7 (46.67)	14 (22.22)	11 (42.31)	5.620	0.122
Drinking, *n* (%)		2 (14.29)	5 (33.33)	14 (22.22)	10 (38.46)	3.953	0.282
Systolic blood pressure (mmHg)		130.71 ± 14.06	146.53 ± 18.70	142.19 ± 20.10	153.35 ± 21.51	4.347	0.006^a,c,f^
Diastolic blood pressure (mmHg)		84.50 (77.00, 86.00)	89.00 (79.00, 102.00)	86.00 (80.00, 92.00)	93.00 (81.00, 101.00)	2.134	0.146
WBC (10^9^/L)		7.52 ± 2.92	6.83 ± 1.84	7.09 ± 1.92	7.06 ± 2.23	0.268	0.848
RBC (10^12^/L)		4.73 ± 0.47	4.57 ± 0.44	4.52 ± 0.42	4.55 ± 0.50	0.861	0.464
PLT (10^9^/L)		267.79 ± 77.59	228.20 ± 70.15	229.02 ± 47.94	228.96 ± 55.92	2.045	0.112
HGB (g/L)		142.77 ± 16.04	137.40 ± 19.00	138.89 ± 12.85	141.30 ± 15.14	0.498	0.684
NUET (%)		0.64 ± 0.13	0.62 ± 0.09	0.63 ± 0.11	0.62 ± 0.11	0.069	0.977
LYMPH (%)		0.29 ± 0.11	0.28 ± 0.08	0.28 ± 0.09	0.28 ± 0.10	0.036	0.991
MONO (%)		0.05 (0.05, 0.08)	0.07 (0.05, 0.08)	0.07 (0.06, 0.08)	0.08 (0.07, 0.09)	11.712	0.008^c^
Fasting glucose (mmol/L)		5.08 (4.62, 5.88)	5.33 (4.67, 5.69)	5.53 (5.09, 6.33)	5.75 (4.97, 6.49)	4.529	0.210
Triglycerides (mmol/L)		1.43 (0.91, 2.52)	1.26 (1.07, 1.56)	1.36 (0.97, 2.06)	1.15 (0.89, 1.44)	2.947	0.400
Total cholesterol (mmol/L)		4.89 ± 0.67	4.94 ± 0.89	4.63 ± 1.02	4.51 ± 1.34	0.780	0.507
HDL-C (mmol/L)		1.17 (1.05, 1.32)	1.17 (1.08, 1.41)	1.13 (1.03, 1.34)	1.08 (1.02, 1.70)	0.338	0.953
LDL-C (mmol/L)		2.73 (2.25, 2.95)	2.51 (2.13, 3.08)	2.25 (1.79, 2.61)	2.09 (1.35, 2.86)	6.506	0.089
HCY (μmol/L)		12.60 (11.25, 13.03)	12.80 (9.70, 15.30)	12.00 (10.5,16.2)	14.20 (12.63, 20.58)	5.100	0.302

NC, normal control; SIVD-NCI, subcortical ischemic vascular disease-normal cognitive impairment; SIVD-VCIND, subcortical ischemic vascular disease-vascular cognitive impairment no dementia; SIVD-VD, subcortical ischemic vascular disease-vascular dementia; WBC, white blood cell count; RBC, red blood cell; PLT, platelet count; HGB, hemoglobin; NUET, neutrophil ratio; LYMPH, lymphocyte ratio; MONO, monocyte ratio; HDL-C, high-density lipoprotein cholesterol; LDL-C, low-density lipoprotein cholesterol; HCY, homocysteine.

*p* values with “^a^” indicate significant differences between HC and SIVD-NCI.

*p* values with “^b^” indicate significant differences between HC and SIVD-VCIND.

*p* values with “^c^” indicate significant differences between HC and SIVD-VD.

*p* values with “^d^” indicate significant differences between SIVD-NCI and SIVD-VCIND.

*p* values with “^e^” indicate significant differences between SIVD-NCI and SIVD-VD.

*p* values with “^f^” indicate significant differences between SIVD-VCIND and SIVD-VD.

### 3.2. A comparison of cognitive function among the four groups of participants

The MoCA, AVLT, STT, and BNT scores were statistically significant difference among the four groups (*p* < 0.05). The scores of MoCA, AVLT, and BNT were lower and the time of STT-B was more prolonged in the SIVD-VCIND group than in the NC group (*p* < 0.05). The MoCA scores were lower and the STT time was more prolonged in the SIVD-VCIND group than in the SIVD-NCI group (*p* < 0.05). The scores of MoCA, AVLT, and BNT were lower and the time on STT was more prolonged in the SIVD-VD group than in the NC and SIVD-NCI groups (*p* < 0.05). The scores of MoCA, AVLT-D, AVLT-R, and BNT were lower and the STT time was more prolonged in the SIVD-VD group than in the SIVD-VCIND group (*p* < 0.05; Table 2).

**Table 2:** Psychopsychological scale characteristics of participants.

	NC (*n* = 14)	SIVD-NCI (*n* = 15)	SIVD-VCIND (*n* = 63)	SIVD-VD (*n* = 26)	*F/H* value	*p* value
MOCA	28.00 (26.00, 28.25)	27.00 (26.00, 28.00)	22.00 (19.00, 23.00)	13.00 (7.00, 17.00)	87.643	<0.001^b,c,d,e,f^
AVLT-I	7.17 (5.92, 8.17)	5.33 (4.33, 6.33)	4.00 (3.33, 5.00)	3.50 (2.58, 4.00)	36.483	<0.001^b,c,e^
AVLT-D	8.25 (6.38, 10.13)	5.50 (3.50, 7,50)	3.50 (2.00, 5.50)	1.50 (0.00, 4.00)	41.466	<0.001^b,c,e,f^
AVLT-R	23.00 (22.00, 24.00)	22.00 (20.00, 23.00)	20.00 (18.00, 22.00)	16.00 (13.75, 19.25)	39.811	<0.001^b,c,e,f^
STT-A(s)	58.00 (46.75, 76.75)	60.00 (45.00, 68.00)	78.00 (61.00, 100.00)	138.50 (112.50, 189.50)	43.960	<0.001^c,d,e,f^
STT-B(s)	126.21 ± 39.45	138.80 ± 47.34	194.19 ± 59.42	301.62 ± 85.71	33.646	<0.001^b,c,d,e,f^
BNT	26.00 (22.00, 27.00)	22.00 (21.00, 25.00)	19.00 (17.00, 22.00)	17.50 (15.00, 21.25)	26.606	<0.001^b,c,e^

NC, normal control; SIVD-NCI, subcortical ischemic vascular disease-normal cognitive impairment; SIVD-VCIND, subcortical ischemic vascular disease-vascular cognitive impairment no dementia; SIVD-VD, subcortical ischemic vascular disease-vascular dementia; MoCA, Montreal Cognitive Assessment; AVLT-I & AVLT-D & AVLT-R, Auditory Verbal Learning Test, immediate recall, delayed recall, and recognition recall; STT-A, Shape trail Test-A; STT-B, Shape Trail Test-B; BNT, Boston Naming Test.

*p* values with “^a^” indicate significant differences between HC and SIVD-NCI.

*p* values with “^b^” indicate significant differences between HC and SIVD-VCIND.

*p* values with “^c^” indicate significant differences between HC and SIVD-VD.

*p* values with “^d^” indicate significant differences between SIVD-NCI and SIVD-VCIND.

*p* values with “^e^” indicate significant differences between SIVD-NCI and SIVD-VD.

*p* values with “^f^” indicate significant differences between SIVD-VCIND and SIVD-VD.

### 3.3. Results of craniocerebral volume analysis among the four groups

**Figure 1:**
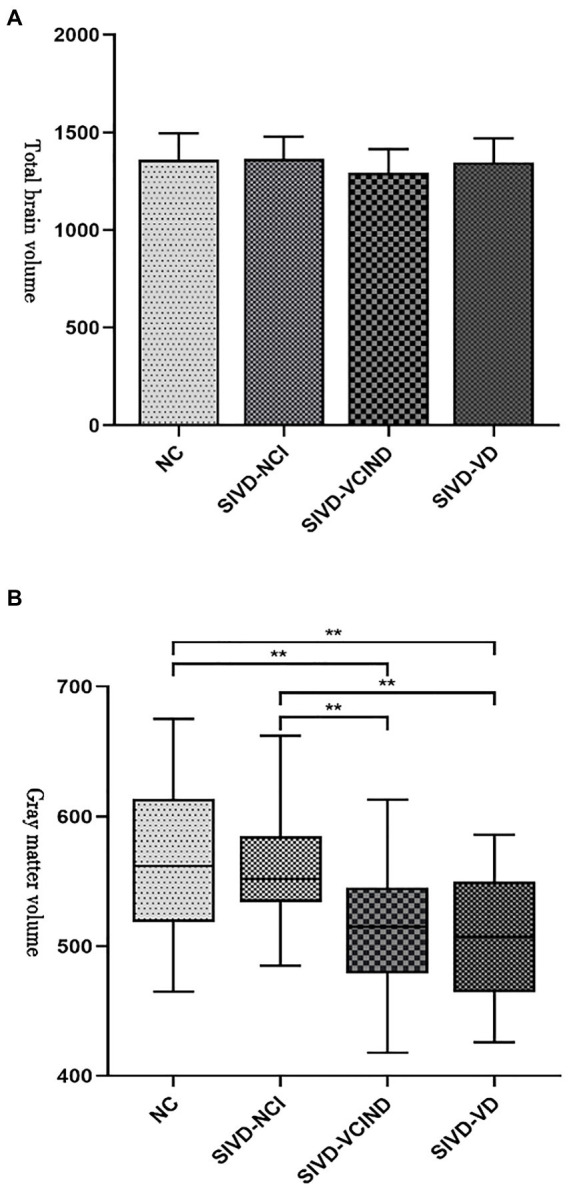
**(A, B)** Analysis results of total craniocerebral and gray matter volumes among the four groups. “^**^” indicate *p* < 0.01.

There was no statistical difference in the total craniocerebral volume among the four groups (*p* > 0.05), but there was a statistically significant difference in the white and gray matter volumes of the craniocerebral brain (*p* < 0.05). The cerebral white and gray matter volumes were lower in the SIVD-VCIND and SIVD-VD groups than in the NC and SIVD-NCI groups (*p* < 0.05; see Table 3; Figures 1A,B for detailed data).

**Table 3:** Structural magnetic resonance characteristics of participants.

	NC (*n* = 10)	SIVD-NCI (*n* = 12)	SIVD-VCIND (*n* = 43)	SIVD-VD (*n* = 16)	*F* value	*p* value
Gray matter volume	564.21 ± 63.63	555.33 ± 45.41	515.35 ± 45.91	503.92 ± 51.21	7.077	<0.001^b,c,d,e^
White matter volume	495.86 ± 56.78	479.27 ± 45.40	440.54 ± 59.22	425.12 ± 46.67	7.077	<0.001^b,c,d,e^
Total brain volume	1,360.93 ± 135.60	1,365.27 ± 113.73	1,294.21 ± 120.64	1,348.15 ± 122.11	2.592	0.056

NC, normal control; SIVD-NCI, subcortical ischemic vascular disease-normal cognitive impairment; SIVD-VCIND, subcortical ischemic vascular disease-vascular cognitive impairment no dementia; SIVD-VD, subcortical ischemic vascular disease-vascular dementia.

*p* values with “^a^” indicate significant differences between HC and SIVD-NCI.

*p* values with “^b^” indicate significant differences between HC and SIVD-VCIND.

*p* values with “^c^” indicate significant differences between HC and SIVD-VD.

*p* values with “^d^” indicate significant differences between SIVD-NCI and SIVD-VCIND.

*p* values with “^e^” indicate significant differences between SIVD-NCI and SIVD-VD.

*p* values with “^f^” indicate significant differences between SIVD-VCIND and SIVD-VD.

### 3.4. Gray matter atrophy analysis among the four groups

**Figure 2:**
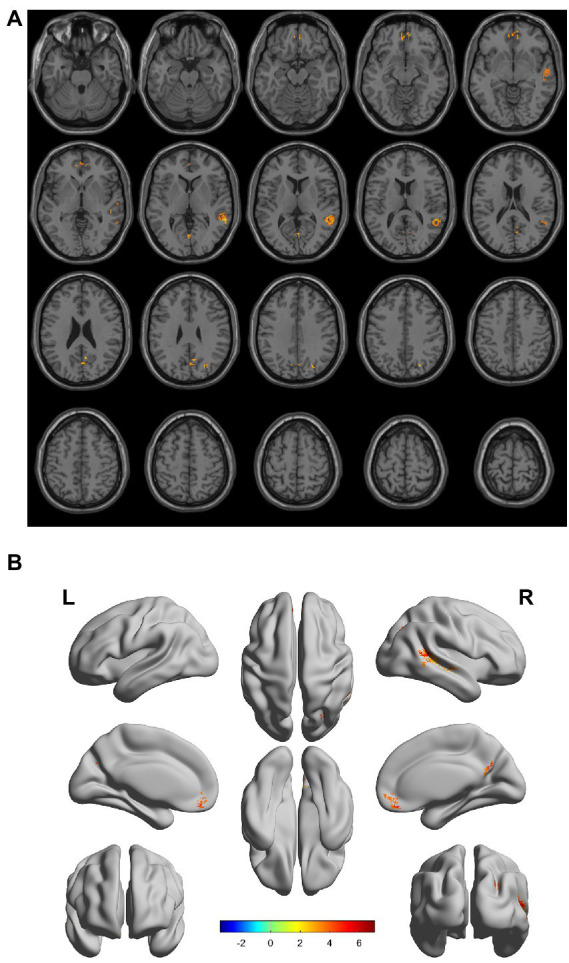
**(A, B)** Compared with the normal control group, the distribution of gray matter atrophy changed in the subcortical ischemic vascular disease-vascular cognitive impairment no dementia group. Orange-yellow shows the areas of difference.

**Figure 3:**
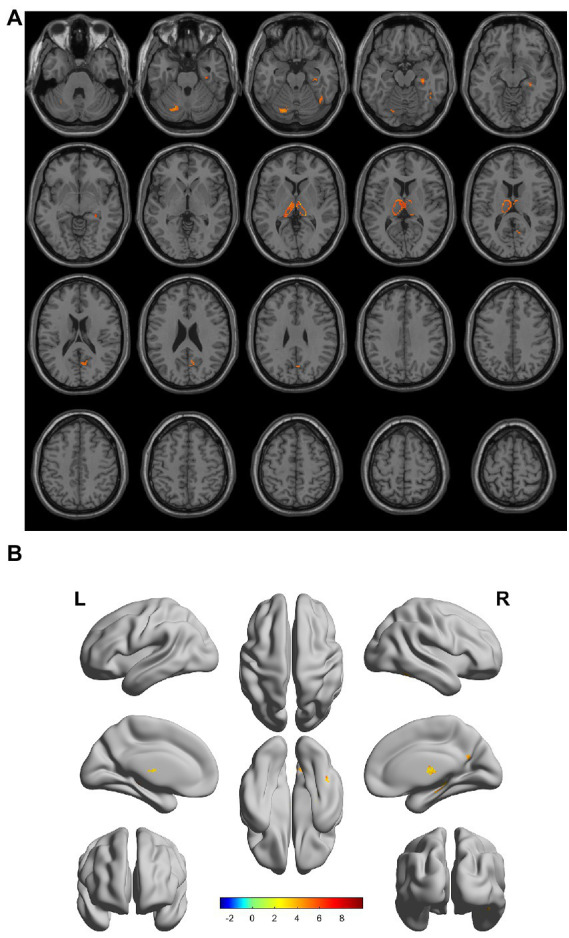
**(A, B)** Compared with the normal control group, the distribution of gray matter atrophy changed in the subcortical ischemic vascular disease-vascular dementia group. Orange-yellow shows the areas of difference.

**Figure 4:**
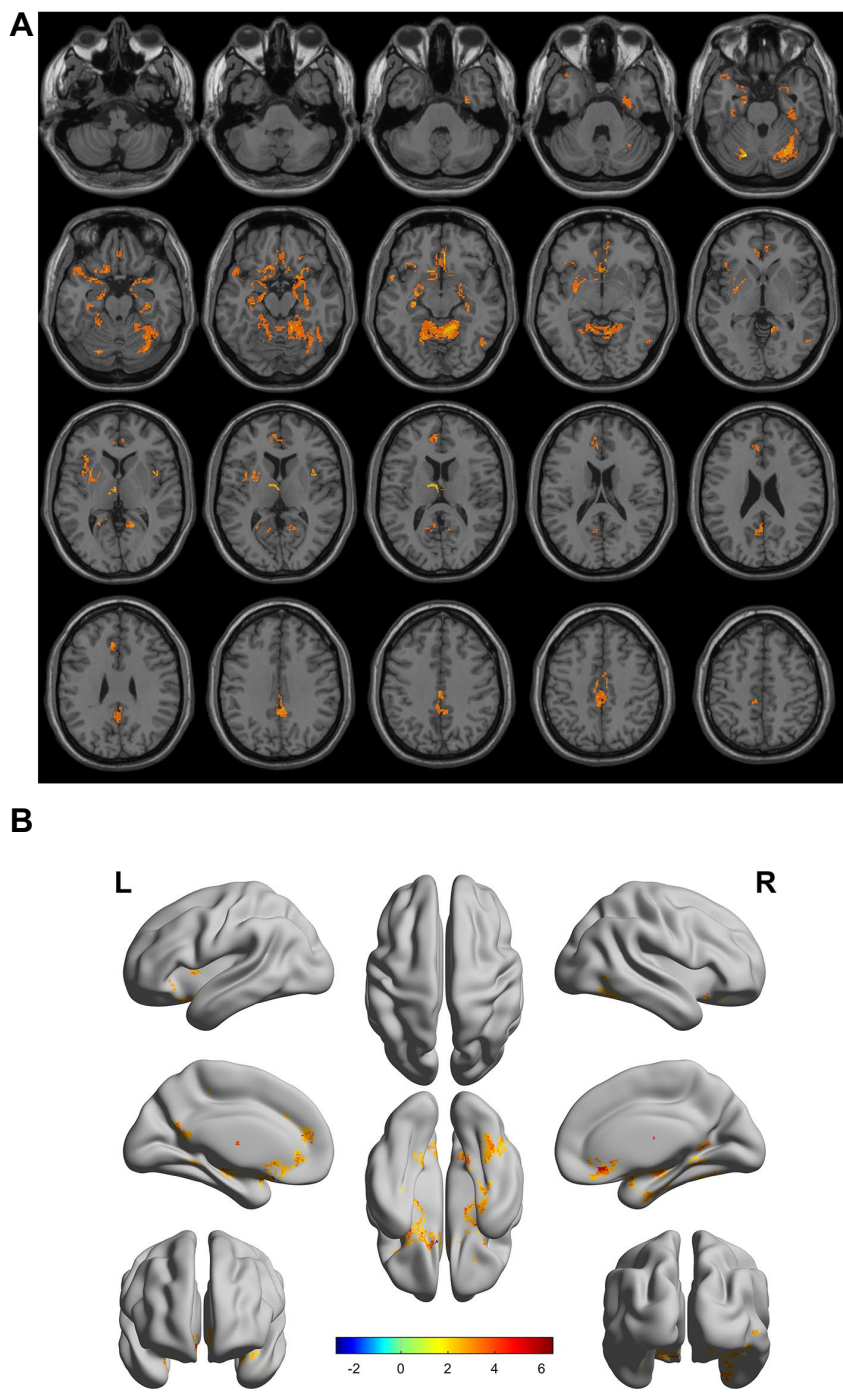
**(A, B)** Compared with the subcortical ischemic vascular disease (SIVD)-normal cognitive impairment group, the area distribution of gray matter atrophy was observed in the SIVD-vascular dementia group. Orange-yellow shows the areas of difference.

**Figure 5:**
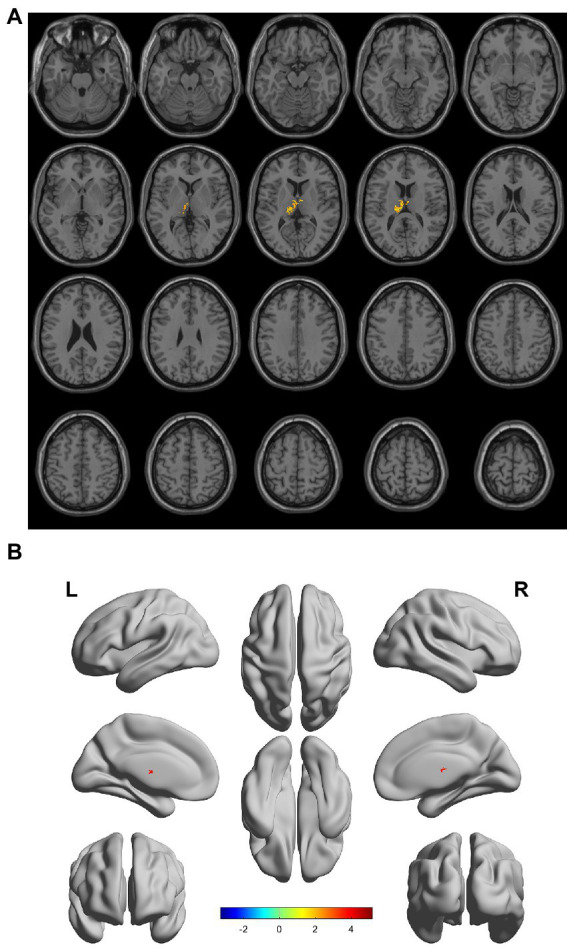
**(A, B)** Compared with the subcortical ischemic vascular disease (SIVD)-vascular cognitive impairment no dementia group, the distribution of gray matter atrophy changed in the SIVD-vascular dementia group. Orange-yellow shows the areas of difference.

There were gray matter atrophy changes observed in the bilateral orbitofrontal gyrus, right middle temporal gyrus, superior temporal gyrus, and precuneus in the SIVD-VCIND group compared to the NC group (*p* < 0.05, family-wise error corrected; see Figures 2A,B for detailed data). Compared with the NC group, gray matter atrophy was observed in the left cerebellar region 6, cerebellar crural region 1, bilateral thalamus, right precuneus, and calcarine in the SIVD-VD group (*p* < 0.05, family-wise error corrected; see Figures 3A,B for detailed data). Compared with the SIVD-NCI group, gray matter atrophy changes were observed in the right cerebellar region 6, cerebellar regions 4–5, left cerebellar region 6, precuneus, thalamus, bilateral median cingulate, and paracingulate gyri in the SIVD-VD group (*p* < 0.05, family-wise error corrected; see Figures 4A,B for detailed data). Compared with the SIVD-VCIND group, gray matter atrophy changes were observed in the bilateral thalamus in the SIVD-VD group (*p* < 0.05, family-wise error corrected; see Figures 5A,B for detailed data; Table 4).

**Table 4:** Comparison of gray matter reduction areas among the four groups (*p* < 0.05, FWE corrected).

	Brain regions (AAL)	Peak MNI coordinate			Number of voxels	Peak *T* value
		*x*	*y*	*z*		
NC vs. SIVD-VCIND	Frontal_Med_Orb_R/Frontal_Med_Orb_L	7.5	43.5	−6	304	5.18
	Temporal_Mid_R/Temporal_Sup_R	60	−43.5	4.5	896	7.03
	Precuneus_R	3	−69	28.5	130	5.17
SIVD-VCIND vs. SIVD-VD	Thalamus_L /Thalamus_R	7.5	−9	15	511	5.17
NC vs. SIVD-VD	Cerebelum_6_L /Cerebelum_Crus1_L	−18	−73.5	−24	363	6.84
	Thalamus_L/Thalamus_R	−7.5	−10.5	16.5	911	9.84
	Precuneus_R/Calcarine_R	9	−63	19.5	143	5.07
SIVD-NCI vs. SIVD-VD	Cerebelum_6_R/Cerebelum_4_5_R	9	−52.5	−9	5,155	6.27
	Cerebelum_6_L	−21	−70.5	−25.5	228	6.48
	Precuneus_L	−16.5	−45	1.5	40	3.70
	Thalamus_L	−12	−10.5	16.5	190	5.72
	Cingulum_Mid_L /Cingulum_ Mid_R	−6	−13.5	45	81	4.35

AAL, automated anatomical labeling; MNI, Montreal Neurological Institute; NC, normal control; SIVD-VCIND, subcortical ischemic vascular disease-vascular cognitive impairment no dementia; SIVD-VD, subcortical ischemic vascular disease-vascular dementia; SIVD-NCI, subcortical ischemic vascular disease-normal cognitive impairment; FWE, family-wise error.

### 3.5. Correlation analysis of region of interest between gray matter volume and neuropsychological scale in the subcortical ischemic vascular disease-vascular cognitive impairment no dementia group

In the SIVD-VCIND group, the total gray matter volume, right superior temporal gyrus, middle temporal gyrus, bilateral medial orbital superior frontal gyrus, and right precuneus were positively correlated with BNT score, whereas the total gray matter volume, right superior temporal gyrus, and middle temporal gyrus were positively correlated with overall cognition (Table 5).

**Table 5:** Correlation analysis of gray matter volume and neuropsychological scale in the region of interest of the subcortical ischemic vascular disease-vascular cognitive impairment no dementia group.

	Gray matter volume	Frontal_Med_Orb_L	Frontal_Med_Orb_R	Precuneus_R	Temporal_Sup_R	Temporal_Mid_R
MOCA	0.174	0.040	0.100	0.184	0.137	0.259^*^
AVLT-I	0.139	0.091	0.168	0.104	0.198	0.195
AVLT-D	0.050	0.084	0.150	0.022	0.031	0.041
AVLT-R	0.064	−0.028	0.043	0.027	0.110	0.021
STT-A (s)	−0.101	−0.088	−0.087	−0.199	−0.150	−0.202
STT-B (s)	−0.067	−0.005	−0.020	−0.163	−0.105	−0.120
BNT	0.476^**^	0.337^**^	0.340^**^	0.375^**^	0.346^**^	0.454^**^

“^*^” indicate *p* < 0.05, “^**^” indicate *p* < 0.01.

MOCA, Montreal Cognitive Assessment; AVLT-I, Auditory Verbal Learning Test-immediate recall; AVLT-D, Auditory Verbal Learning Test-delayed recall; AVLT-R, Auditory Verbal Learning Test-recognition recall; STT-A, Shape Trail Test-A; STT-B, Shape Trail Test-B; BNT, Boston Naming Test.

## 4. Discussion

In this study, we investigated the changes in gray matter atrophy patterns in patients with SIVD-VCIND. The results showed that the total volume of gray matter decreased in both patients with SIVD-VCIND and SIVD-VD, and gray matter atrophy was observed in the bilateral orbital superior frontal gyrus, right middle temporal gyrus, superior temporal gyrus, and precuneus. With worsening cognitive impairment, gray matter atrophy was observed in the cerebellum and thalamus. In the SIVD-VCIND group, the total gray matter volume, right superior temporal gyrus, middle temporal gyrus, precuneus, and bilateral medial orbital superior frontal gyrus were positively correlated with BNT score, whereas the total gray matter volume, right superior temporal gyrus, and middle temporal gyrus were positively correlated with overall cognition. These results provide insights into the association between structural imaging changes induced by SIVD-VCIND and cognitive impairment, provide valuable evidences to explain the pathogenesis of subcortical VCI, and contribute to the early diagnosis of SIVD-VCIND and early warning of SIVD-VD.

In normally aging older adults without cognitive impairment, the association between larger total WML volume and lower perceptual speed was independent of other cognitive domains and total gray matter volume (Arvanitakis et al., 2016). Wong et al. showed that the VCI scale score was independently correlated with WML volume and global brain and hippocampal atrophy measurements (Wong et al., 2015). This reflects the changes in the underlying brain volume involved in cognitive dysfunction in VCI. Our study found that the total gray matter volume among the four groups changed as follows: NC > SIVD-NCI > SIVD-VCIND > SIVD-VD. This indicates that gray matter atrophy is more extensive in the SIVD-VD, whereas the atrophy degree of SIVD-VCIND is lower than that of SIVD-VD. Patients with SIVD-NCI had reduced gray matter volume, despite no perceived cognitive impairment. This indicates that total gray matter volume is correlated with VCI. Therefore, we should identify and treat SIVD-VCIND as early as possible in clinical practice to reduce the change in gray matter volume caused by VCIND to VD.

The temporal lobe is responsible for language and auditory perception and is involved in long-term memory and emotion (Chen L. et al., 2020). The orbitofrontal cortex is a major component of the prefrontal/subcortical circuit, and disruption of this circuit is associated with executive dysfunction (Wang et al., 2019). The precuneus contains extensive/extensively connected cortical and subcortical structures. As the precuneus is a major associated region, it may have several behavioral functions (Cavanna and Trimble, 2006), and its cognitive functions involve episodic memory, visual–spatial, self-related information processing, metacognition, consciousness, and other processes (Ye et al., 2019). Studies have shown that (Chen et al., 2020) compared with the NC group, the cortical thickness of the bilateral insula, middle temporal gyrus, and right inferior temporal gyrus in the SIVD-NCI was significantly reduced. The thicknesses of the bilateral insula, precuneus, middle inferior temporal gyrus, medial temporal lobe, right middle temporal gyrus, orbital extra side, and posterior central gyrus were significantly reduced in the SIVD-VCI group. Jin Thong et al. (2014) demonstrated extensive gray matter volume atrophy in patients with SIVD-VCIND, especially in the frontal cortex and subcortical regions. In addition, Seo et al. found thinning of the cortex in many regions, including the orbitofrontal cortex and superior temporal gyrus in SIVD-VCIND. In addition to these regions, SIVD-VD also involves the dorsolateral prefrontal and temporal cortices (Seo et al., 2010). In addition, functional MRI studies have shown that with the aggravation of cognitive impairment in patients with SIVD, the efficiency of functional connectivity in the orbitofrontal, parietal, and temporal cortex and basal ganglia decreases (Sang et al., 2018). The nodal efficiency of the prefrontal and temporal cortices decreased more in patients with SIVD-VD than in those with SIVD-VCIND.

The thalamus is the most important subcortical structure in the brain and is associated with almost all cortical brain regions and processes and transmits information from different brain regions. The thalamus regulates several basic cognitive functions, including motivation, motor control, and sensory input processing (Mai and Majtanik, 2018). Because the thalamus acts as a relay station for the extensive circuit connecting gray matter and white matter, it may be considered a candidate center for distinguishing SIVD from Alzheimer’s disease. Studies have isolated diffusion tensor imaging of the thalamus region and found that cell damage in the thalamus region may be greater in patients with SIVD than in those with Alzheimer’s disease, indicating the important role of the thalamus and its connection to the frontal cortex (Tu et al., 2021). Other studies have shown that the prethalamic nucleus, dorsal nucleus, and papillary somatothalamic tract are responsible for the activity of cortical regions during memory and are closely related to episodic memory^[79]^. Therefore, thalamic involvement may be associated with memory impairments. Recently, there is evidence that the cerebellum is involved in processes related to cognitive, behavioral, and mental disease control (Sui and Zhang, 2012). Furthermore, the fact that the cerebellum is interconnected with various limbic structures, including the hippocampus, and the cerebral cortex, including the prefrontal region, provides strong neuroanatomical evidence to support the involvement of the cerebellum in cognitive regulation. The success of drug and psychological interventions in the treatment of cognitive impairment is associated with improvements in cerebellar function (Konarski et al., 2005). Although there are few studies on the correlation between the cerebellum and cognition, this study provides evidence that cerebellar cortical atrophy can cause SIVD-VD. Our results are consistent with those of previous studies, and we found varying degrees of gray matter atrophy in both patients with SIVD-VCIND and SIVD-VD. SIVD-VCIND was mainly concentrated in the frontal and temporal lobes and precuneus cortex. In the SIVD-VD group, gray matter atrophy in the cerebellum and thalamus was the primary manifestation. The atrophy degree of subcortical gray matter structure is closely related to the severity of VCI. Thus, early detection of the atrophy characteristics of subcortical gray matter structure of SIVD-VCIND and treatment can delay the development of VD, and early detection of atrophy of the thalamus and cerebellum and early intervention can delay the further aggravation of VD.

In the SIVD-VCIND group, the total gray matter volume, right superior temporal gyrus, middle temporal gyrus, precuneus, and bilateral medial orbitofrontal superior frontal gyrus were significantly positively correlated with BNT score. BNT is one of the most commonly used nomenclature methods in neuropsychological assessment (Rabin et al., 2016), which mainly represents language dysfunction and is related to frontotemporal lobe damage. The temporal lobe can reflect semantic knowledge storage; therefore, damage to the temporal lobe can affect the associative priming effect. Word naming disorders caused by temporal lobe damage may be related to selective attention, decreased inhibition, and word fluency. In a study on gray matter and language fluency (Martins et al., 2021), children with better verbal fluency had larger gray matter volumes in the right superior temporal gyrus and occipitotemporal, parietal temporal, and fusiform regions. Currently, SIVD-VCIND is still considered to cause serious impairment of the subcortical circuitry and executive function. However, we did not find a significant correlation between RIO gray matter volume and executive function. Therefore, we should evaluate the cognitive impairment of patients in a multidimensional manner through psychological scales and imaging and identify potential patients with SIVD-VCIND as early as possible through language fluency to delay the development of the disease.

This study has some limitations. Because the participants came from a single center and were limited by regional economy and culture, it was difficult for the participants to cooperate with additional auxiliary examinations, such as lumbar puncture collection of cerebrospinal fluid. Therefore, our current study was only based on structural imaging and clinical studies and lacked positron emission tomography, cerebrospinal fluid, and other biomarkers. In addition, the number of disease sources in this study was limited, the sample size was small, and all of which were from the Han population in northeast China. At the same time, due to the large potential population of SIVD-VCIND, this caused the number of our participants to be imbalanced between the groups. There may be selection bias of participants, and this result is not completely applicable to other regions, races, or large-sample studies. This study was retrospective and lacked follow-up data to support the disease outcomes. In terms of methods, we only selected sMRI to analyze the gray matter volume, and there was a lack of comprehensive analysis of functional MRI, including diffusion tensor imaging for the direction of white matter cellulose and spectral magnetic resonance for brain metabolism. Therefore, the combination of structural and functional MRI in patients with SIVD-VCIND will be of more significance in a large-sample prospective study to determine the changes in brain structure and function.

## 5. Conclusion

In conclusion, our study shows that sMRI can detect extensive and subtle structural changes in the gray matter of patients with SIVD-VCIND and SIVD-VD, providing valuable evidences to explain the pathogenesis of subcortical VCI and contribute to the early diagnosis of SIVD-VCIND and early warning of SIVD-VD.

## Data availability statement

The raw data supporting the conclusions of this article will be made available by the authors, without undue reservation.

## Ethics statement

The studies involving human participants were reviewed and approved by The ethics committee of Hongqi Hospital affiliated with Mudanjiang Medical College. The patients/participants provided their written informed consent to participate in this study.

## Author contributions

LT, WZ, and CY contributed to conception and design of the study. JX and JZ collected MRI data. ZW and XD organized the database. LT, RL, and JW performed the statistical analyses and wrote the first draft of the manuscript. All authors contributed to manuscript revision, read, and approved the submitted version.

## Funding

This article was supported by Basic scientific research projects of provincial colleges and universities in Heilongjiang Province (2022-KYYWFMY-0001), the special Scientific Research Plan for duate teacher of Mudanjiang Medical University (8227052581), the applied technology research and development plan project of Mudanjiang Science and Technology Bureau (SQ2022NS076) and the Scientific research project of Heilongjiang Provincial Health Commission (20220303070632).

## Conflict of interest

The authors declare that the research was conducted in the absence of any commercial or financial relationships that could be construed as a potential conflict of interest.

## Publisher’s note

All claims expressed in this article are solely those of the authors and do not necessarily represent those of their affiliated organizations, or those of the publisher, the editors and the reviewers. Any product that may be evaluated in this article, or claim that may be made by its manufacturer, is not guaranteed or endorsed by the publisher.

## Footnotes

^1^ https://www.nitrc.org/projects/mricron/

^2^ http://www.fil.ion.Ucl.ac.uk/spm/

^3^ http://rfmri.org/dpabi

## References

[ref1] Arvanitakis Z. Fleischman D. A. Arfanakis K. Leurgans S. E. Barnes L. L. Bennett D. A. (2016). Association of white matter hyperintensities and gray matter volume with cognition in older individuals without cognitive impairment. Brain Struct. Funct. 221, 2135–2146. doi: 10.1007/s00429-015-1034-7, PMID: 25833685 PMC4592368

[ref2] Biessels G. J. (2016). Diagnosis and treatment of vascular damage in dementia. Biochim Biophys. Mol. Basis Dis. 1862, 869–877. doi: 10.1016/j.bbadis.2015.11.009 26612719

[ref3] Cavanna A. E. Trimble M. R. (2006). The precuneus: a review of its functional anatomy and behavioural correlates. Brain 129, 564–583. doi: 10.1093/brain/awl004 16399806

[ref4] Chen L. Song J. Cheng R. Wang K. Liu X. He M.. (2020). Cortical thinning in the medial temporal lobe and Precuneus is related to cognitive deficits in patients with subcortical ischemic vascular disease. Front. Aging Neurosci. 12:614833. doi: 10.3389/fnagi.2020.614833, PMID: 33679368 PMC7925832

[ref5] Chen Q. Wang Y. Qiu Y. Wu X. Zhou Y. Zhai G. (2020). A deep learning-based model for classification of different subtypes of subcortical vascular cognitive impairment with FLAIR. Front. Neurosci. 14:557. doi: 10.3389/fnins.2020.00557, PMID: 32625048 PMC7315844

[ref6] Frantellizzi V. Pani A. Ricci M. Locuratolo N. Fattapposta F. de Vincentis G. (2020). Neuroimaging in vascular cognitive impairment and dementia: a systematic review. J. Alzheimers Dis., 73, 1279–1294, doi: 10.3233/JAD-191046. 31929166

[ref7] Iadecola C. (2013). The pathobiology of vascular dementia. Neuron 80, 844–866. doi: 10.1016/j.neuron.2013.10.008 24267647 PMC3842016

[ref8] Jin Thong J. Y. du J. Ratnarajah N. Dong Y. Soon H. W. Saini M.. (2014). Abnormalities of cortical thickness, subcortical shapes, and white matter integrity in subcortical vascular cognitive impairment. Hum. Brain Mapp. 35, 2320–2332. doi: 10.1002/hbm.22330, PMID: 23861356 PMC6869364

[ref9] Konarski J. Z. McIntyre R. S. Grupp L. A. Kennedy S. H. (2005). Is the cerebellum relevant in the circuitry of neuropsychiatric disorders? J. Psychiatry Neurosci. 30, 178–186. 15944742 PMC1089778

[ref10] Le Couteur D. G. Wahl D. Naismith S. L. (2017). Comorbidity and vascular cognitive impairment-no dementia (VCI-ND)[J]. Age Ageing 46, 705–707. doi: 10.1093/ageing/afx080 28481963

[ref11] Mai J. K. Majtanik M. (2018). Toward a common terminology for the thalamus. Front. Neuroanat. 12:114. doi: 10.3389/fnana.2018.00114 30687023 PMC6336698

[ref12] Martins M. Reis A. M. Castro S. L. Gaser C. (2021). Gray matter correlates of reading fluency deficits: SES matters, IQ does not. Brain Struct. Funct. 226, 2585–2601. doi: 10.1007/s00429-021-02353-1, PMID: 34357437

[ref13] Nasreddine Z. S. Phillips N. A. Bédirian V. Charbonneau S. Whitehead V. Collin I.. (2005). The Montreal cognitive assessment, MoCA: a brief screening tool for mild cognitive impairment. J. Am. Geriatr. Soc. 53, 695–699. doi: 10.1111/j.1532-5415.2005.53221.x 15817019

[ref14] Rabin L. A. Paolillo E. Barr W. B. (2016). Stability in test-usage practices of clinical neuropsychologists in the United States and Canada over a 10-year period: a follow-up survey of INS and NAN members. Arch. Clin. Neuropsychol. 31, 206–230. doi: 10.1093/arclin/acw007 26984127

[ref15] Rockwood K. Wentzel C. Hachinski V. Hogan D. B. MacKnight C. McDowell I. (2000). Prevalence and outcomes of vascular cognitive impairment. Vascular cognitive impairment investigators of the Canadian study of health and aging[J]. Neurology 54, 447–451. doi: 10.1212/WNL.54.2.447 10668712

[ref16] Román G. C. Erkinjuntti T. Wallin A. Pantoni L. Chui H. C. (2002). Subcortical ischaemic vascular dementia. Lancet Neurol. 1, 426–436. doi: 10.1016/S1474-4422(02)00190-4 12849365

[ref17] Roman G. C. Tatemichi T. K. Erkinjuntti T. Cummings J. L. Masdeu J. C. Garcia J. H.. (1993). Vascular dementia: diagnostic criteria for research studies. Report of the NINDS-AIREN international workshop. Neurology 43, 250–260. doi: 10.1212/wnl.43.2.250, PMID: 8094895

[ref18] Sachdev P. Kalaria R. O'Brien J. Skoog I. Alladi S. Black S. E.. (2014). Diagnostic criteria for vascular cognitive disorders: a VASCOG statement. Alzheimer Dis. Assoc. Disord. 28, 206–218. doi: 10.1097/WAD.0000000000000034, PMID: 24632990 PMC4139434

[ref19] Sang L. Chen L. Wang L. Zhang J. Zhang Y. Li P.. (2018). Progressively disrupted brain functional connectivity network in subcortical ischemic vascular cognitive impairment patients. Front. Neurol. 9:94. doi: 10.3389/fneur.2018.00094, PMID: 29535678 PMC5834750

[ref20] Seo S. W. Ahn J. Yoon U. Im K. Lee J. M. Tae Kim S.. (2010). Cortical thinning in vascular mild cognitive impairment and vascular dementia of subcortical type. J. Neuroimaging 20, 37–45. doi: 10.1111/j.1552-6569.2008.00293.x, PMID: 19220710

[ref21] Smith E. E. (2017). Clinical presentations and epidemiology of vascular dementia. Clin. Sci. 131, 1059–1068. doi: 10.1042/CS20160607 28515342

[ref22] Sui R. Zhang L. (2012). Cerebellar dysfunction may play an important role in vascular dementia. Med. Hypotheses 78, 162–165. doi: 10.1016/j.mehy.2011.10.017 22075237

[ref23] Tu M. C. Huang S. M. Hsu Y. H. Yang J. J. Lin C. Y. Kuo L. W. (2021). Discriminating subcortical ischemic vascular disease and Alzheimer's disease by diffusion kurtosis imaging in segregated thalamic regions. Hum. Brain Mapp. 42, 2018–2031. doi: 10.1002/hbm.25342, PMID: 33416206 PMC8046043

[ref24] Wang J. Liang Y. Chen H. Wang W. Wang Y. Liang Y.. (2019). Structural changes in white matter lesion patients and their correlation with cognitive impairment. Neuropsychiatr. Dis. Treat. 15, 1355–1363. doi: 10.2147/NDT.S194803, PMID: 31190839 PMC6534061

[ref25] Wong A. Wang D. Black S. E. Nyenhuis D. L. Shi L. Chu W. C. W.. (2015). Volumetric magnetic resonance imaging correlates of the National Institute of Neurological Disorders and Stroke-Canadian stroke network vascular cognitive impairment neuropsychology protocols. J. Clin. Exp. Neuropsychol. 37, 1004–1012. doi: 10.1080/13803395.2015.1038983, PMID: 26332179

[ref26] Wu X. P. Gao Y. J. Yang J. L.. (2015). Quantitative measurement to evaluate morphological changes of the corpus callosum in patients with subcortical ischemic vascular dementia. Acta Radiol. 56, 214–218. doi: 10.1177/0284185114520863 24445093

[ref27] Ye Q. Zou F. Dayan M.. (2019). Individual susceptibility to TMS affirms the precuneal role in meta-memory upon recollection. Brain Struct. Funct. 224, 2407–2419. doi: 10.1007/s00429-019-01909-6 31254060

